# Construction Notes

**Published:** 1893-04-22

**Authors:** 


### CONSTRUCTION NOTES.

The new buildings of the Chesterfield Hospital are approaching completion, but in spite of the impetus which the Duke of Devonshire gave to the fund for defraying the cost by his donation of £500, the total sum promised up to the end of last year was only £3,453. In consequence of the erection of this new wing, two small wards in the old building have been thrown out of use, the consequent result being that the space for the in-patients is much restricted.

The freeholders of the common lands of Rotherham have signified their intention of adding a children's ward to the existing hospital building. The new ward will be called the "Feofee's" (or Freeholder's) Ward, by whom the whole cost of the erection will be borne. In view to the future maintenance of this extension, it is proposed to raise money through a general Sunday-school collection which presumably will bring in about £50. The internal arrangements of the hospital have been much improved this year, and more has been done for the nursing and comfort of the patients.

A newly-furnished ward to the Clayton Hospital was formally opened on Wednesday, March 22nd. This ward, which gives an extra accommodation for 10 beds, had hitherto been reserved, but now, owing to the pressure of increasing inmates, it has been prepared for use. The furnishing has cost, together with the requisite nurses' room, £1°0, whilst the ordinary expenditure of maintenance and other charges will entail an increased annual expenditure from £450 to £500. The opening of this new ward will necessitate that further sleeping accommodation be provided for nurses and servants, to provide which will involve a further expenditure of £250.

The alterations, which are fast approaching completion, at the Bristol Eye Hospital include the enlargement of the waiting-room, the provision of accommodation for the house-surgeon, and the building of a new ward-room. The principal improvement is, however, the provision of a large new ward, capable of containing 12 extra beds, so that if the income of the charity allow it, 28 beds may now be established in the institution. The piece of ground at the back of the building is being laid out as a garden. The sanitary arrangements have been entirely remodelled on the latest and most approved methods. The managing committee have been content to modify and improve the existing building rather than to enter upon the great expense which the construction of a new hospital would entail. The cost of the enlargement has been about £3,000, of which £800 remains to be raised.

Extensive improvements and alterations have taken place at York County Hospital in connection with the sanitary arrangements. A new and efficient laundry has been added, and the kitchens have been reconstructed. The heating of the corridors and wards is consequently much improved. The need of improved bed-rooms for the nurses having constantly occupied the attention of the committee, an alteration has been effected by changing the large infectious wards, and thus all the bed-rooms are grouped together into one building. Two small infectious wards have been added on to the eye ward, and separated from the main building.

The Rural Sanitary Authority's Emergency Hospital at Rumworth for the treatment of small-pox and typhus cases in the Bolton district was formally opened the second week in March. Messrs. Morton and Co., of Liverpool, who were entrusted with the building, have satisfactorily carried out the work. The dimensions of the new hospital are 75 feet in length and 20 in width. It is divided into two wards, each of which contains six beds, and immediately between those wards, and opposite the main entrance, is the nurses room; at the end of the building are lavatories and baths, the whole forming a very compact arrangement, and the building is admirably planned; the total cost of which, including the furnishing, will be about £500. In the absence of small-pox the hospital will possibly be used as a resort for patients in the Fever Hospital who are convalescent.

